# Malaria Hyperendemicity and Risk for Artemisinin Resistance among Illegal Gold Miners, French Guiana

**DOI:** 10.3201/eid2205.151957

**Published:** 2016-05

**Authors:** Vincent Pommier de Santi, Félix Djossou, Nicolas Barthes, Hervé Bogreau, Georges Hyvert, Christophe Nguyen, Stéphane Pelleau, Eric Legrand, Lise Musset, Mathieu Nacher, Sébastien Briolant

**Affiliations:** Military Center for Epidemiology and Public Health, Marseille, France (V. Pommier de Santi);; Direction Interarmées du Service de Santé en Guyane, Cayenne, French Guiana (V. Pommier de Santi, F. Djossou, N. Barthes, H. Bogreau, G. Hyvert, C. Nguyen, S. Briolant);; Andrée Rosemon Hospital, Cayenne (F. Djossou, M. Nacher);; Institut de Recherche Biomédicale des Armées, Brétigny-sur-Orge, France (H. Bogreau, C. Nguyen, S. Briolant);; Institut Pasteur de la Guyane, Cayenne (H. Bogreau, C. Nguyen, S. Pelleau, E. Legrand, L. Musset, S. Briolant);; World Health Organization Collaborative Center for Surveillance of Antimalarial Drug Resistance, National Resistance Center of Malaria, Cayenne (S. Pelleau, E. Legrand, L. Musset);; Institut Pasteur, Genetic and Genomics of Insects Vectors, Paris (E. Legrand);; Centre d'Investigation Clinique INSERM 1424 and EA3593 Université de Guyane, Cayenne (M. Nacher)

**Keywords:** malaria, French Guiana, illegal gold mining, hyperendemicity, epidemiology, vector-borne infections, parasites, antimicrobial resistance, artemisinin, *Suggested citation for this article*: Pommier de Santi V, Djossou F, Barthes N, Bogreau H, Hyvert G, Nguyen C, et al. Malaria hyperendemicity and risk for artemisinin resistance among illegal gold miners, French Guiana. Emerg Infect Dis. 2016 May [*date cited*]. http://dx.doi.org/10.3201/eid2205.151957

## Abstract

To assess the prevalence of malaria among illegal gold miners in the French Guiana rainforest, we screened 205 miners during May–June 2014. Malaria prevalence was 48.3%; 48.5% of cases were asymptomatic. Patients reported self-medication with artemisinin-based combination therapy. Risk for emergence and spread of artemisinin resistance among gold miners in the rainforest is high.

Malaria control programs on the Guiana Shield, a region of South America, are challenged by migrant populations looking for gold. Since 2008, the “Harpie” operation to control and reduce illegal gold mining activities has been conducted by French Armed Forces in French Guiana. Military deployments at illegal gold mining sites have resulted in several outbreaks and increased incidence of malaria in French forces (*1*–*4*), which suggests high transmission levels in those areas. Illegal gold mining sites are isolated places in the rainforest, far from health posts. The miners are usually hidden in the forest to avoid police controls, and they live in unsanitary conditions.

Although formal health monitoring is not carried out in these communities, the effects of infectious diseases are of concern. In 2013, a group of 34 illegal gold miners with severe diarrheic and respiratory symptoms were evacuated by plane from a health post to the reference regional hospital in Cayenne. The outbreak was attributed to co-infection with several parasitic, bacterial, or viral agents: seasonal influenza A(H1N1)pdm09, *Shigella flexnieri*, *Necator americanus*, *Leishmania* spp., *Streptococcus pneumoniae*, and *Plasmodium vivax* (*5*). All patients came from the illegal gold mining site of Eau Claire (3°36′00′′N, 53°34′60′′W) (Figure 1). Given these problems, French health authorities decided to provide primary medical care in the field and also to assess the sanitary situation in Eau Claire. We describe the results of the cross-sectional study conducted to assess the epidemiologic situation of malaria.

**Figure 1:**
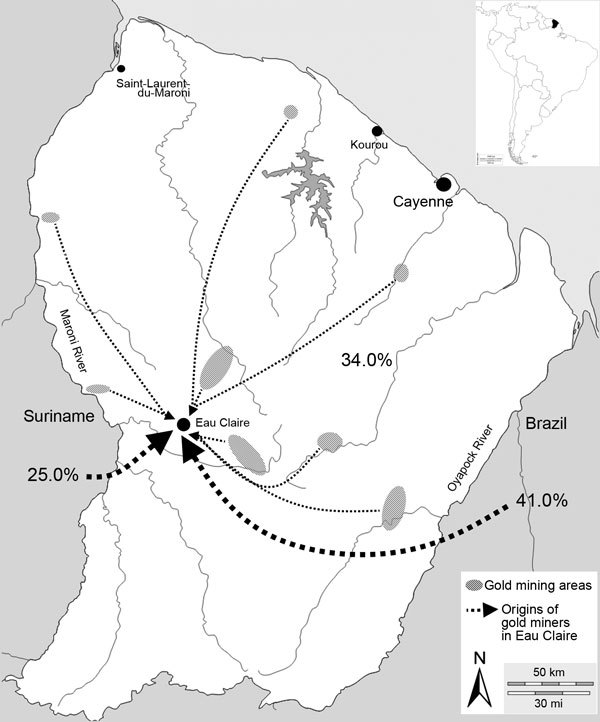
Origins of gold miners (N = 205) before they began to work in the illegal gold mining site of Eau Claire, French Guiana. Inset shows location of French Guiana in South America.

## The Study

Because of violence and insecure conditions at illegal mining sites, French military health services conducted the mission. Twelve soldiers and military policemen accompanied the medical team to ensure their protection but also that of the miners’ community. A primary care clinic and laboratory were set up under tents at the Eau Claire gold mining camp from May 28, 2014, through June 6, 2014. Active malaria screening was offered to every person who sought care for any reason. 

Diagnostic tests associating the malaria rapid diagnosis test (RDT) (SD Bioline Malaria Ag *Pf*/Pan; Standard Diagnostics, Inc., Giheung-gu, South Korea) and thin blood films were performed in the field. Patients who had positive results of an RDT, thin blood film, or both received treatment. 

Data were collected by physicians concerning each person’s recent medical history, protection measures against mosquito bites, use of medications, and recent travel inside or outside of French Guiana. Patient anonymity was stringently respected; every patient was issued a unique identification number. Only verbal consent could be obtained to avoid references that might reveal the identity of undocumented persons engaged in illegal activities. 

Dried blood spots were obtained on filter paper by fingerstick and packaged in individual plastic bags with a desiccant until processing. *Plasmodium* DNA was extracted subsequently and tested with a nested PCR targeting *P. falciparum* and *P. vivax* 18S rRNA genes, according to the method of Snounou et al. (*6*). The propeller domain of *pfK13* gene was sequenced in *P. falciparum* isolates (*7*).

We defined* Plasmodium* infection as a positive RDT, thin blood film, or PCR result. Symptomatic *Plasmodium* infection was defined as a positive test result and fever (history of fever in preceding 24 hours and/or documented temperature >38°C during medical examination) and/or >2 of the following: nausea, vomiting, diarrhea, abdominal pain, anorexia, headache, or jaundice. Other *Plasmodium* infections were classified as asymptomatic.

**Figure 2:**
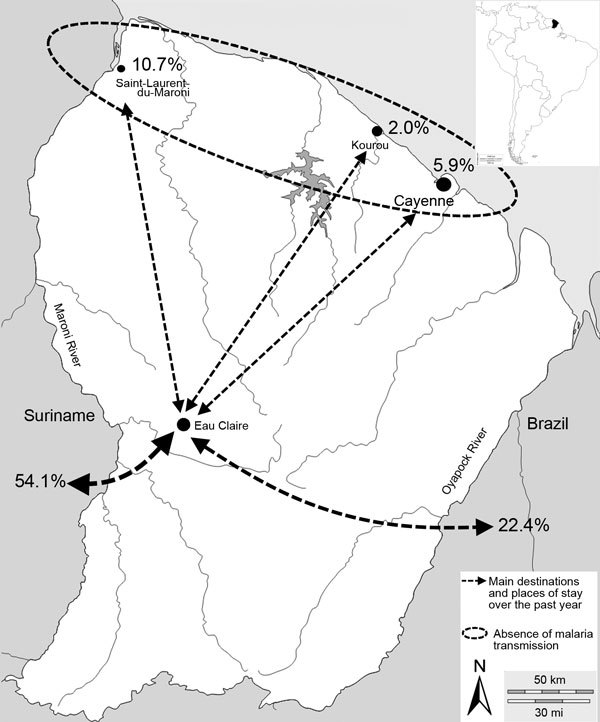
Travels of gold miners (N = 205) living in Eau Claire, French Guiana, 2013–2014. Inset shows location of French Guiana in South America.

Overall, 205 persons freely sought medical care and accepted malaria screening. The sex ratio was 2.0 (137 [66.8%] men; 68 [33.2%] women). Median age was 39 years (interquartile range [IQR] 32–46 years; range 20–63 years). The workers had been gold panning for a median time of 7 years (IQR 3–14 years; range 1 month–44 years) and on illegal gold mining sites in French Guiana for a median time of 4 years (IQR 1–8 years; range 1 month–25 years). Most (97.6%, 200) patients came from Brazil, 4 (2.0%) came from Suriname, and 1 (0.5%) came from Guyana. Before their arrival at Eau Claire, patients had lived in Brazil (41.0%, 84), Suriname (24.9%, 51), or at another illegal gold mining site (19/69 [33.7%] different sites throughout inland French Guiana and 1 unknown site) (Figure 1). During the previous year, 66.0% (135/205) persons had traveled to >1 area outside Eau Claire: 54.1% (111/205) to Suriname (among those, 60.0% had traveled there >2 times); 22.4% (46/205) to Brazil; and 18.5% (38/205) to the main cities in the malaria-free area along the French Guiana coast (Figure 2).

Of the 205 patients, 156 (76.1%) reported >1 fever episode and 131 (63.9%) reported several (2–>4) episodes; 121 (59.0%) reported >1 malaria episode and 93 (45.4%) several episodes (Table 1). Self-medication with antimalarial drugs was reported by 120 (58.5%) patients, of whom 118 (98.3%) had reported malaria episodes in the past year. Artemisinin-based combination therapies (ACTs) were mainly used: dihydroartemisinin/piperaquine/trimethoprim (Artecom; Chongqing Tonghe Pharmaceutical Co. Mingshan Town, China) by 79 (63.7%) and artemether-lumefantrine (Coartem; Novartis Pharmaceuticals Corp. Basel, Switzerland) by 32 (26.7%). Chloroquine was also used alone or with ACTs by 13 (10.8%) patients. The medication schedules used were not clearly identified, but 53 (44.2%) patients took drugs for 1 or 2 days only. Nets were used by 37 (18.0%) and mosquito repellents by 41 (20.5%) of the 205 patients. 

**Table 1:** Malaria and fever episodes reported by illegal gold miners, French Guiana, 2013–2014*

Episodes	No. (%) patients, N = 205
Malaria episode	
No	84 (41.0)
Yes	121 (59.0)
No. episodes	
1	27 (22.3)
2–4	48 (39.7)
>4	45 (37.2)
NA	1 (0.8)
Fever episode	
No	46 (22.4)
Yes	156 (76.1)
NA	3 (1.5)
No. episodes	
1	23 (14.8)
2–4	74 (47.4)
>4	57 (36.5)
NA	2 (1.3)

*NA, no answer.

The overall prevalence of malaria infection was 48.3% (99/205). *P. falciparum* and *P. vivax* single infections accounted for 44.4% (44/99) and 29.3% (29/99) cases, respectively, and mixed infection with *P. falciparum* and *P. vivax* for 26.3% (26/99) (Table 2). RDTs, thin blood films, and PCR were positive for 40.4% (40/99), 32.3% (32/99), and 97.0% (96/99) of patients classified as positive for malaria, respectively. Asymptomatic infections accounted for 48.5% (48/99) of cases. Low parasitemia levels were systematically observed. Only 1 person had a parsitemia level >1%, and no differences in level were found between symptomatic and asymptomatic infections. The propeller region of the *pfK13* gene was successfully sequenced in 26 *P. falciparum* isolates without any mutation detected.

**Table 2:** Number of positive parasite carriers by *Plasmodium* species according to diagnostic method, French Guiana, 2013–2014

Test	No. (%) single infections, n = 73	No. (%) mixed *P. falciparum/P. vivax* infections, n = 26	No. (%) total infections, n = 99	% Prevalence/ test, N = 205

*P. falciparum*, n = 44	*P. vivax*, n = 29
RDT*	23 (52.3)	8 (27.6)	8 (30.8)	40 (40.4)	19.5
Thin blood film	16 (36.4)	10 (34.5)	6 (23.1)	32 (32.3)	15.6
RDT and thin blood film	14 (31.8)	8 (27.6)	4 (15.4)	26 (26.3)	12.7
Cumulative RDT/thin blood film	25 (56.8)	10 (34.5)	10 (38.5)	46 (46.5)	22.4
PCR	42 (95.5)	28 (96.6)	26 (100.0)	96 (97.0)†	46.8†

*Malaria rapid diagnostic test: SD Bioline Ag *Pf*/pan (Standard Diagnostics, Inc., Giheung-gu, South Korea). †In 3 cases (2 *P. falciparum* infections, 1 *P. vivax* infection) PCR results were negative, but results of thin blood films were positive.

## Conclusions

During the 10 days of field work, almost all inhabitants of the gold mining site sought medical care. The high prevalence of malaria and asymptomatic *Plasmodium* infections observed confirms that malaria is hyperendemic there (*8*). Because of the mobility of gold miners within French Guiana, malaria also could be highly prevalent among persons at other illegal gold mining sites where competent vectors exist (*3*). This hypothesis is strengthened by the recurrent malaria outbreaks experienced by French forces involved in operations to control illegal gold mining, particularly in the center of the region (*3*). 

Systematic self-medication by patients using ACTs without following a full course of treatment is a serious risk for emergence of resistance to artemisinin and associated drugs (*9*). The high price of ACTs in the field (2 g gold) was the primary reason patients gave for not completing treatments. The mobility of gold miners raises 2 issues: the reintroduction of the disease in malaria-free areas (*10*) and the spread of antimalarial drug resistance if the disease emerges (*11*). In 2013, parasite persistence on day 3 after treatment with Coartem was described in Suriname, and most participants in that study had worked in gold mines in French Guiana (*12*,*13*). In Guyana, *pfK13* C580Y mutants obtained from samples in 2010 were recently reported (*14*). These results may be viewed as ominous. Collaboration between countries of the Guiana Shield to control malaria among mobile populations is urgently needed (*15*).
